# Periscope

**Published:** 1882-05

**Authors:** 


### PERISCOPE.

*U. S. Medical Investigator*, January 15th.—Dr. Cranch writes: "In the retention and suppression of urine that often occur in fevers, Sepia, in the 30th or 200th dilution has done most excellent service, in restoring the secretion to its normal character. It is indicated by delay in voiding urine, after the desire is felt, along with scanty, muddy discharge, the sediment of which *sticks to the vessel*, and the *odor* of which is abominable. If these symptoms are *not* present, some other drug than Sepia must be exhibited; notably, Lycopodium, Apis, Belladonna or Opium, in high dilutions."

March 1st.—Dr. Jones is quoted as giving the following: "The vomiting of Stramonium is grass green, aggravated by raising the head [nausea, Bry.] and at times by light."

A few somewhat similar symptoms suggest themselves. Jahr gives the following remedies with vomiting aggravated by motion:

COLCH. Vomiting renewed by every movement.

IPEC. Vomiting on stooping.

TABAC. Vomiting commences as soon as he begins to stir about.

OPIUM. Disposition to vomit when moving and after eating.

KALI B. Vomiting on moving about.

STRAM. Jahr gives: vomiting of bile after least motion, such as sitting up in bed.

SILICEA, has nausea after any exertion which raises the temperature of the body.

THERIDION, nausea on rising in morning; on motion.

VERAT. Vomiting whenever he moves or drinks.

ZINC. Nausea, with vomiting and retching, renewed by least motion.

*Hahnemannian Monthly*, January.—Dr. Laird reports a case of diarrhœa cured by Berberis; patient had diarrhœa for a year and a half. Had been under homœopathic and allopathic treatment. At time of prescribing Berberis, patient had from two to six painless, watery clay-colored, offensive stools a day. Stools preceded by pain about navel and accompanied by emission of fetid flatus, occasionally having involuntary stools during sleep, but as a rule, the diarrhœa begins in the morning after rising and ceases by night. Exercise of any kind—standing, riding, walking or even long-continued conversation—causes a decided aggravation. Complexion sallow; especially marked when stools are few, and partially disappears as they increase in number. Felt a weak, gone feeling in stomach and abdomen; worse from exercise or talking. *Soreness and tenderness of the renal region, aggravated by the least jar or pressure; tearing pains in back, extending down the ureters and shooting into hips.* Sleep, restless, disturbed by dreams. Feeling of weakness and general *malaise.* Berberis [2c], night and morning, cured in a week. The medicine was prescribed chiefly upon its characteristic renal symptoms, as above italicized.

Dr. Farrington writes on Moschus. He says, "the most important hysterical symptoms of musk are: attacks even to fainting or unconsciousness; coldness of the surface; pale face; suffocative spasms; scolding, until she falls unconscious.

Compare Castoreum, Nux mosch., Asafœtida, Ammoniac, Valerian, Ignatia, Magnesia muriatica.

Camphor antidotes many of its symptoms, especially if unconscious and coldness are present.

Castoreum is derived from the preputial sacs of the beaver. Like musk, it causes nervousness, twitching and deranged menses. But it is more adapted to the nervous symptoms which precede fully-developed hysteria. It suits women who suffer from irritable weakness, abdominal symptoms predominating. * * *

Castoreum, exhausted, pains better from pressure; menstrual colic with pallor and cold sweat.

Nux moschata, errors of perception, drowsy; faints; enormous tympany; oppression of heart to throat; skin dry, cool.

Valeriana, nerves irritated, cannot keep still; tearings, cramps, better when moving; taste of tallow or slimy.

Asafœtida, reverse peristalsis, rancid eructations, offensive flatus; tightness of the chest; checked discharges.

Magnesia muriatica, faints at dinner, relief from eructations; head better from pressure and wrapping up; palpitation better on moving about; stools crumble.

Moschus has been employed by allopathic physicians, when, in the course of pneumonia, a purely nervous delirium obtains. The brain is violently excited, patient talks nonsense with furious vivacity. (Trousseau.)

